# Disturbance of desire‐goal motivational dynamics during different exercise intensity domains

**DOI:** 10.1111/sms.14129

**Published:** 2022-01-27

**Authors:** Ian M. Taylor, Summer Whiteley, Richard A. Ferguson

**Affiliations:** ^1^ School of Sport, Exercise & Health Sciences, Loughborough University Leicestershire United Kingdom

**Keywords:** desire‐goal conflict, exercise domains, lactate threshold, motivation, self‐control

## Abstract

**Purpose:**

The desire‐goal motivational conflict helps explain endurance performance; however, the physiological concomitants are unknown. The present study examined disturbances in desire to reduce effort and performance goal value across moderate, heavy, and severe exercise intensity domains, demarcated by the first (LT1) and second (LT2) lactate thresholds. In addition, the within‐person relationships among blood lactate concentration, heart rate, and desire‐goal conflict were examined.

**Methods:**

Thirty participants (53% female, *M_age_* = 21.03 years; *SD* = 2.06 years) completed an incremental cycling exercise test, in which work rate was increased by 25 watts every four minutes, until voluntary exhaustion or sufficient data from the severe intensity domain had been collected. Desire to reduce effort, performance goal value, blood lactate concentration (for determination of LT1 and LT2), and heart rate were measured at the end of each stage and analyzed using multilevel models.

**Results:**

The desire to reduce effort increased over the exercise test with additional shifts and accelerations after each lactate threshold. The performance goal did not show general declines, nor did it shift at LT1. However, the performance goal value shifted at LT2, and the rate of change increased at both thresholds. Within‐person variation in blood lactate concentration positively correlated with the desire to reduce effort and negatively correlated with the performance goal. Within‐person variation in heart rate correlated with desire to reduce effort but not the performance goal.

**Conclusion:**

Transitioning through both lactate thresholds is important phases for motivation during progressive exercise, particularly for the desire to reduce effort. Within‐person variation in blood lactate concentration is more influential for motivation, compared with heart rate.

## 1 INTRODUCTION

The ability to endure discomfort is critical in sport and many other areas of human performance, such as the military and survival in extreme circumstances. It is unsurprising, therefore, that endurance is a popular topic of investigation in the sport, exercise, and performance sciences. Having been dominated by physiological perspectives, our understanding of human endurance has benefited recently from researchers integrating psychological and physiological perspectives to offer more comprehensive models of human endurance.^1^, ^2^ Building on this trend, the present study investigates the potential physiological underpinnings of motivational processes during an endurance task. Rather than defining endurance in terms of time or distance, as is done in applied contexts, we refer to an endurance act as requiring persistence in the face of psychological and physiological difficulties. This definition, therefore, can be applied to many scenarios beyond “endurance” classified events (e.g., 10 000 m athletics event).

Endurance in performance contexts requires considerable tolerance and management of psychophysiological discomfort. Afferent signals (i.e., sensory impulses transferred from parts of the body to the central nervous system) associated with physiological responses (including those associated with exercise) are affectively labeled^3^ and integrated into a collective representation of the current physiological condition compared with homeostasis.^4^ This depiction subsequently emerges as a single conscious, motivational state,^5^ which becomes increasingly aversive as a function of exercise intensity.^6^ Hence, a desire to reduce effort will evolve because humans have a proclivity to avoid discomfort^7^ and maintain homeostasis.^8^ This proximal desire vies with the distal goal of successful performance. In endurance settings, the content of the goal may vary, for example, winning a race, achieving a pre‐specified time, or exerting a specific amount of effort during the task. This content is less important, compared with the motivational strength of the goal and its conflict with the desire to reduce effort. Such desire‐goal conflicts represent a central aspect of all self‐control dilemmas^9^ and provide an empirically supported framework to investigate endurance performance.^10^, ^11^ For example, lower desire to reduce effort at the beginning of a cycling time trial and slower reductions in goal importance across the trial have been found to be characteristic of better performance^12^ Measures of the desire and goal value at the midpoint of a cycling trial also predicted cycling performance at a high intensity.^12^

Within this desire‐goal conflict model of endurance, it is assumed that the underlying basis for the desire to reduce effort is hedonic and stems from basic drives to maintain homeostasis.^10^ This implies that the desire to reduce effort may have significant physiological underpinnings. In contrast, the value of the performance goal is primarily underpinned by internal (e.g., improvement and personal value) and external (squad selection, prize money) incentive structures, and physiological responses to exercise are less influential. Nonetheless, this assumption remains to be empirically tested until now.

Broadly speaking, the physiological foundation of motivation during endurance acts is not a new area of research. Endeavors have typically focused on the relationship between physiological responses to exercise and perceived exertion or effort.^2^ Responses include heart rate, oxygen uptake, respiratory rate, and blood lactate concentration, yet no single parameter consistently explains feelings of exertion.^13^ The psychobiological model of endurance proposes that afferent signals from physiological perturbances have little motivational value because they are independent of perceived effort.^1^ This body of work, therefore, implies that physiological responses to exercise may have little influence on motivational processes during endurance. By focusing on perceived effort; however, an incomplete portrayal of motivation and its physiological concomitants is presented. Physiological responses to exercise and their generalized core affective labels (i.e., states that vary simply on pleasantness and activation) are motivationally salient because they form the basis of desires that are often contrary to valued goals.^14^, ^15^ Indeed, the central purpose of affect associated with afferent bodily signals is to motivate action.^4^, ^16^ The desire‐goal conflict is a motivational framework that can assimilate this psychophysiological knowledge.

The physiological response to exercise varies as a function of the intensity at which it is performed.^17^ These responses have been characterized into exercise intensity domains,^17^, ^18^ which are delineated by specific physiological thresholds. The moderate intensity domain refers to intensities below the so‐called first lactate threshold, defined as the intensity after which there is a sustained increase in blood lactate concentration above resting values (LT1).^19^, ^20^ This domain is characterized by a steady state cardiopulmonary response and little or no sustained increase in blood lactate concentration. The heavy intensity domain refers to intensities above LT1, but below critical power, which is analogous to the so‐called second lactate threshold when there is a second rise in blood lactate concentration above resting levels (LT2).^21^ This domain is characterized by a delayed steady state and emergence of a V̇O_2_ slow‐component, which eventually stabilizes after 20–30 minutes, as well as a sustained but gradual increase in blood lactate concentration. During severe domain exercise (at intensities above LT2), no steady state is achieved, V̇O_2_ progresses to reach V̇O_2max,_ and blood lactate concentration increases progressively. Evaluation of these domains is possible via a progressive exercise test in which blood lactate concentration is regularly measured. This allows estimation of the two primary physiological (lactate) thresholds that delineate the moderate‐heavy, and heavy‐severe boundaries, respectively, as well as the subsequent progression through each of the domains. As such, these two thresholds represent an opportunity to analyze disruptions to the desire‐goal conflict at points when broad metabolic and cardiopulmonary system responses to exercise become unstable.

Complementary to inspecting specific thresholds and domains, it is worth establishing whether the desire‐goal conflict is sensitive to continuous changes in metabolic and cardiopulmonary responses over the course of an endurance act. Comparing findings from the two approaches will establish whether the desire‐goal conflict is sensitive to general micro‐fluctuations (i.e., within‐person variation) in physiological motivational inputs or stronger macro‐fluctuations (i.e., lactate thresholds) are required to disrupt the desire‐goal conflict. In addition to repeated measurements of blood lactate concentration as a metabolic response to exercise, cardiopulmonary responses are also likely to influence motivational states.^22^ Heart rate, for example, is known to produce interoceptive information that informs emotional states.^23^, ^24^ During treadmill exercise, heart rate, unlike other respiratory factors (e.g., V̇O_2_ and respiratory exchange ratio), remains stable when perceptions of effort are fixed,^25^ albeit the relationship between HR and effort is likely correlational rather than causal.^13^ Collectively, this research implies that heart rate may be an underlying input to motivational states during exercise.

In sum, recent work has established the desire‐goal conflict as an important framework to study human endurance.^10^, ^11^, ^12^ The present study aims to build on this work by investigating the physiological concomitants of the desire‐goal conflict during progressive exercise. It was hypothesized that the trajectories of the desire to reduce effort and performance goal value would be disrupted when participants transition through relevant physiological thresholds from the moderate to heavy intensity domain (hypothesis 1a and 1b), and from heavy to severe intensity domain (hypothesis 2a and 2b). Specifically, the desire to reduce effort was expected to shift and/or accelerate positively, whereas the performance goal value was expected to shift and/or accelerate negatively. Moreover, it was hypothesized that within‐person variation in blood lactate and heart rate would positively predict the desire to reduce effort (hypothesis 3a and 3b) and negatively predict the performance goal value (hypothesis 4a and 4b).

## 2 MATERIALS & METHODS

### 2.1 Participants

Thirty participants (14 males, 16 females, *M_age_ *= 21.03 years; *SD* = 2.06 years) were recruited through a university scheme in which students can participate in studies for course credit, as well as adverts placed with university triathlon and cycling teams. Participants were required to be 18–35 years old, physically active (i.e., a minimum of 30 minutes moderate intensity activity three days a week for three months) and free of pre‐existing medical conditions or family history that made high intensity exercise potentially unsafe. Sample size targets were based on a minimum of 30 level‐2 units (i.e., participants in the present study) required for minimal bias in statistical parameters, when combined with at least five level‐1 units (i.e., measurement points in the present study) for multilevel modeling.^26^

### 2.2 Procedure

All experimental procedures were approved by a university ethics approvals committee and conformed with the Declaration of Helsinki. Participants were fully informed of study details and the risks and discomforts associated with all experimental trials. It was clarified that participation was voluntary, data would be stored anonymously, and they had a right to withdraw at any point during the study without consequences. Participants provided written informed consent and completed questionnaires to establish that they met the inclusion criteria (i.e., they were generally healthy and physically active). Participants were initially instructed that the goal of the session was to place as high as possible on a leaderboard containing all participants’ performance scores and, to enhance the meaningfulness of the goal, they were to set a target position on the leaderboard. Leaderboards are commonly used to set meaningful goals in psychology research, especially when the goal is difficult.^27^

Participants performed an incremental cycling test on an electronically braked cycle ergometer (Lode Excalibur Sport, Lode B.V. Gronigen, The Netherlands). Ergometer saddle and handlebar dimensions were setup to suit individual specifications. Participants cycled at a freely chosen pedal rate at an initial work rate of either 50 or 100 watts (W) depending on personal preference and experience (e.g., cyclist/triathlete). This flexibility was permitted because we were not interested in performance data (e.g., time to exhaustion), only underlying psychological and physiological data during exercise. Work rate was increased by 25 W at four‐minute intervals. Visual information regarding pedal rate and workload was obscured to avoid participants using this information to regulate their performance. During the third minute of each four‐minute stage, participants were presented with measures of their desire to reduce effort and performance goal value. During the last thirty seconds of each stage, capillary blood samples were obtained for the immediate measurement of blood lactate concentration. Heart rate was recorded at the end of each stage. The test continued until voluntary exhaustion or when two stages had been completed in which a clear increase in blood lactate concentration above 4 mmol.L^−1^ had occurred (to obtain data in the severe intensity domain), whichever came first. We did not ask participants to continue beyond two stages in the severe domain because this was not necessary to answer the research questions.

### 2.3 Measures

***Desire and goal value***. The desire to reduce effort was measured by verbal responses to the instruction “Please rate to what extent do you want to reduce your effort” on a 20‐point scale, ranging from 1 (not wanting to reduce effort at all) to 20 (definitely want to reduce effort immediately). The value of the performance goal was measured by responding to the instruction “Please rate how important is it to achieve your goal” on a 20‐point scale, ranging from 1 (not important at all) to 20 (extremely important). Similar scales have demonstrated predictive and nomological validity in previous work.^12^

***Blood lactate concentration*, *lactate thresholds*, *and establishment of exercise domains***. Capillary blood samples were taken from the earlobe and immediately analyzed for blood lactate concentration (Lactate Pro 2, Arkray, Japan). A blood lactate/work rate curve was modelled for each participant using publicly available software.^28^ The work rate corresponding to an initial increase of 1 mmol.L^−1^ above baseline concentration during the initial stage of the exercise test, and fixed blood lactate concentration of 4 mmol.L^−1^ were defined as LT1 and LT2, respectively, and were used to demarcate the moderate, heavy, and severe domains of exercise. Blood lactate concentration is a reliable method to determine exercise intensity,^29^ generally superior to heart rate^30^ and circumvents the need to assess expired gases for V̇O_2_.

***Heart rate***. Heart rate was continually monitored (T31 transmitter and FT1 watch, Polar Electro Oy, Kempele, Finland).

### 2.4 Data analysis

MLwiN software (version 3.05^31^) was used to construct multilevel models to test study hypotheses. This method was used because of the hierarchical structure of the data with each measurement of desire, goal value, blood lactate concentration, and heart rate (Level‐1 time‐varying units) nested within each participant (Level‐2 units^32^). First, unconditional means models (i.e., no predictor variables) were formed to describe the variance of study variables associated with level‐1 (i.e., within‐person) and level‐2 errors (i.e., between‐person). To test hypothesis 1 and 2, two multilevel growth models (for desire and goal value, respectively) were constructed by simultaneously adding a linear time predictor variable (each time point was coded as 1, 2, 3, etc.), two dichotomous “threshold” predictor variables indexing pre‐(coded as 0) to post‐(coded as 1) LT1 and LT2 threshold measures, respectively. In addition, two higher order interaction terms between each threshold variable and linear time were included. These models estimate a) the degree of linear change in desire and goal value over the course of the trial, b) the change in mean levels as a function of the respective thresholds, and c) the alterations in rate of change as a function of the thresholds.

To examine hypotheses 3 and 4, two multilevel models without time and threshold variables included, but with lactate concentration and heart rate as predictor variables of desire and goal value. The predictor variables were centered around each participant's unique mean (i.e., group mean centered), therefore, the models estimated whether within‐person variation (as opposed to individual differences) in blood lactate or heart rate predicted desire to reduce effort and performance goal value.

## 3 RESULTS

### 3.1 Descriptive Statistics

Descriptive statistics for the study variables at LT1 and LT2 can be found in Table 1. On average, participants completed 7.57 stages (*SD* = 1.74), which are equivalent to approximately 30 minutes of work. Twenty‐eight participants cycled until voluntary exhaustion, and two participants were stopped twice by an investigator because two stages in the severe intensity domain had been completed. Average heart rate at LT1 and LT2 was 83 percent and 91 percent, respectively, of participants’ heart rate at task cessation. Average workload at LT1 and LT2 was 68 percent and 81 percent, respectively, of participants’ peak workload. Unconditional means models revealed that 20 percent of the variance in performance goal value was attributable to within‐person variation, and 80 percent attributable to individual variation. In contrast, 85 percent of the variance in desire to reduce effort was attributable to within‐person variation, and 15 percent was attributable to individual variation.

**TABLE 1:** Descriptive statistics of study variables

Variable	Lactate Threshold 1		Lactate threshold 2	
	Mean (*SD*)	Range	Mean (*SD*)	Range
Desire to reduce effort	4.67 (3.60)	1–15	7.04 (4.80)	1–18
Performance goal value	14.67 (4.55)	3–20	14.22 (4.76)	3–20
Heart rate (beats.min−1)	151 (14)	119–175	165 (14)	133–186
Power (Watts)	155.56 (58.56)	75–300	184.26 (56.82)	75–325

### 3.2 Trajectories of desire to reduce effort and performance goal value across intensity domains (hypotheses 1 and 2)

**FIGURE 1:**
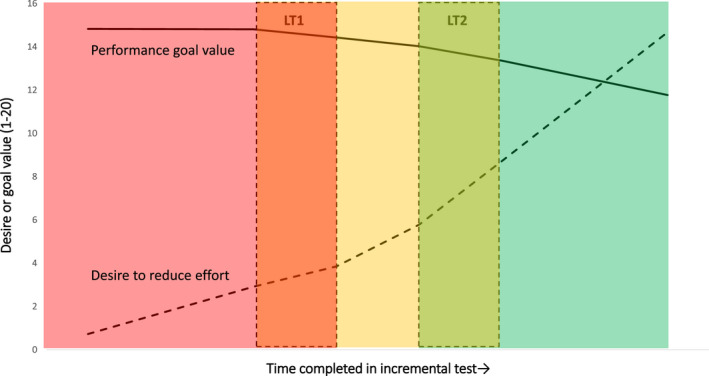
Illustrative trajectories of desire and performance goal value during the cycling trial

In Table 2, multilevel growth model 1 describes the trajectory of the desire to reduce effort over the trial, therefore, investigating hypotheses 1a and 2a. The model indicated that the desire to reduce effort generally increased over the course of the trial (linear time coefficient), shifted at LT1 and LT2 (threshold coefficients), and accelerated after each threshold (interaction terms). This pattern in desire to reduce effort is illustrated in Figure 1, which describes predicted trajectories in desire and goal value over time and when participants transition through LT1 and LT2. Variance terms indicated that the linear increase and the shift at the LT2 varied in magnitude across individuals, but the shift at the LT1 did not. When allowing the interaction terms to vary across individuals the model did not converge; hence, these parameters are not reported.

**TABLE 2:** Multilevel growth models describing desire to reduce effort and performance goal value across LT1 and LT2 thresholds

Outcome	Desire to reduce effort (model 1)	Goal value (model 2)
*Fixed Effects (SE in parentheses)*		
Intercept	−0.37 (0.37)	**14.84 (0.99)**
Linear time	**1.09 (0.21)**	0.01 (0.14)
LT1 threshold	**−3.50 (0.99)**	1.21 (0.80)
Time ×LT1 threshold	**0.84 (0.20)**	**−0.40 (0.16)**
LT2 threshold	**−5.26 (1.34)**	**2.08 (1.01)**
Time ×LT2 threshold	**1.04 (0.22)**	**−0.39 (0.18)**
*Variance Terms*		
Intercept	1.20 (0.84)	**27.92 (7.49)**
Linear time	**0.85 (0.30)**	**0.39 (0.13)**
LT1 threshold	1.21 (1.34)	
Time ×LT1 threshold		
LT2 threshold	**5.31 (2.40)**	1.76 (1.03)
Time ×LT2 threshold		

Note

Bold figures indicate statistical significance (*p* ≤ .05). Exact values can be calculated from the *Z* scores (*b*/*SE* for the fixed effects). Variance terms are not reported in instances when the predictor variable was not permitted to vary across individuals to aid convergence of models. LT = Lactate Threshold.

Model 2 describes the trajectory of performance goal value over the trial, therefore, investigating hypotheses 1b and 2b. The model revealed that the performance goal value did not linearly decline over the course of the trail (linear time coefficient), nor did it shift at LT1 (threshold coefficient). However, the performance goal value shifted at LT2 (threshold coefficient), and the rate of change was disturbed at both thresholds (interaction terms). This pattern in goal value across the course of the trial is illustrated in Figure 1. Variance terms indicated that the linear change, but not the shift at LT2, significantly varied across individuals. When allowing the remaining effects to vary across individuals, the model did not converge; hence, these parameters are not reported.

### 3.3 Within‐person variation of blood lactate concentration and heart rate predicting desire and goal value (hypotheses 3 and 4)

Multilevel models revealed that within‐person variation in blood lactate concentration positively correlated with the desire to reduce effort (*b* = 0.69, *p* < .001) and negatively correlated with the performance goal value (*b* = −0.15, *p* = .02). A small positive correlation between within‐person variation in heart rate and desire to reduce effort (*b* = 0.05, *p* < .001) was observed, but no significant relationship between heart rate and performance goal value (*b* = 0.00, *p* = .37).

## 4 DISCUSSION

The desire‐goal conflict has been proposed as a valid framework to study motivational dynamics during endurance performance,^10^ but potential underpinning physiological concomitants were unknown until now. Two different blood lactate thresholds were inspected to observe changes in the desire to reduce effort and value of a performance goal at these periods, which are characterized by physiological instability. Moreover, within‐person variation in blood lactate concentration and heart rate was examined as correlates of the desire and performance goal value. Results indicated that progression from the moderate through to the heavy and severe domains of exercise by transitioning through LT1 and LT2, respectively, are points in which motivational dynamics are perturbed, particularly the desire to reduce effort. Variation in blood lactate concentrations was a stronger correlate of the desire to reduce effort and performance goal value, compared with variation in heart rate.

In any activity requiring self‐control, the desire to stop will increase as a function of time.^33^ Unsurprisingly, therefore, a general increase in the desire to reduce effort was observed in the present study. As expected, however, this desire deviated from its trajectory and began to accelerate when the LT1 occurred, and participants entered the heavy exercise domain. The same picture is presented regarding LT2 and entry into the severe exercise domain. At this point, the desire to reduce effort shifts again and further accelerates. Previous research has not modeled changes in motivational factors as a function of these two physiological thresholds. Nonetheless, core affect has been shown to be stable below the ventilatory threshold (which typically occurs at a similar exercise intensity as the LT1^13^) but become increasingly negative after it.^34^ Our results align with the idea that hedonic motivational factors that encourage avoidance of unpleasant states, such as negative affect and desire to reduce effort, become increasingly powerful when aversive physiological responses accumulate. The causal pathway linking physiological responses and generalized core affective labels,^3^ which subsequently manifest into a motivational state,^5^ were not investigated here but seems the most plausible explanation for this pattern of findings.

Both physiological thresholds also have implications for the value of performance goal. The value of the goal did not shift at LT1; however, after a period of stability it began to significantly decline at this point. LT2 (and entry into the severe intensity domain) coincided with a shift in level and a change in rate of decline. The smaller regression coefficients compared with the desire to reduce effort imply that underlying physiological responses contribute less to the performance goal value. Nonetheless, it may be inaccurate to suggest that the performance goal value is entirely underpinned by more stable reflective factors, such as internal and external incentives.^10^ The lactate thresholds may signify that goal achievement is becoming increasingly difficult, therefore, reductions in goal value may occur to protect self‐worth (i.e., defensive pessimism^35^). The comparable (albeit in opposing directions) disturbances of desire and goal value at the two lactate thresholds imply that they are not isolated motivational components with distinct concomitants, but they share some physiological correlates during progressive exercise.

The primary aim of some types of endurance training is to delay reaching the lactate thresholds and associated physiological consequences resulting in fatigue. The collective findings of the present study imply that achieving this aim also has important motivational ramifications. These physiological thresholds are associated with increases in the motivational potency of the desire to reduce effort and simultaneous decreases in the value of the performance goal. As such, the relative weight of each motivational component shifts in favor of the proximal desire, and diminished endurance performance occurs.^12^ Positive motivational consequences can, therefore, be added to the list of benefits resulting from delayed lactate thresholds.

In addition to the examination of physiological thresholds and intensity domains, the results demonstrated that within‐person variation in blood lactate concentration was positively associated with the desire to reduce effort and negatively associated with the performance goal value. The relationship was stronger with the desire to reduce effort, compared with the performance goal value, again implying that blood lactate concentration is more salient for the hedonic desire to reduce effort than the performance goal value. A relatively smaller positive correlation between within‐person variation in heart rate and desire to reduce effort was observed, and no significant relationship between heart rate and performance goal value. Hence, the magnitude of the regression coefficients suggests that the relationship between lactate and motivation is stronger than heart rate and motivation. Previous work offers no definitive conclusion regarding the relative coupling of heart rate and lactate with motivational factors. Some evidence suggest perceived exertion is correlated to a greater extent with heart rate compared with lactate concentrations.^36^ However, the present analysis focuses on within‐person variation, rather than absolute levels, which makes comparison with previous research difficult. It is likely that deviations of physiological state within an individual are more likely to stimulate motivational responses (within‐person variation), as opposed to individual differences leading to corresponding differences in motivation (between‐person differences). The impact of lactate concentration on motivational factors is likely not direct, of course, but indirect via pH changes associated with lactic acid production.^37^ The affective sensation associated with this process may be more potent, given that heart rate interoceptive signals can be easily suppressed in favor of alternative stimuli.^38^

### 4.1 Limitations and Future Research Directions

Future research should experimentally manipulate underpinning physiological states to establish causal effects on the desire‐goal conflict. This can be achieved acutely through, for example, nutritional supplementation such as prior ingestion of sodium bicarbonate or ammonium chloride to induce metabolic alkalosis or acidosis, respectively,^39^ or chronically through exercise training. A familiarization trial would be necessary to include in this type of experiment, which did not occur in the present study. In addition, a measure of affect alongside the desire to reduce effort would provide a more detailed examination of the assumed relationship between affective responses and the desire to reduce effort. Third, our healthy and active sample may limit the generalizability of some of the findings in the study. For example, participants with experience of strenuous activity may appraise the associated sensations of lactate accumulation less negatively, compared with an unhealthy and inactive sample.

The prevailing method of analyzing physiological responses to exercise is to treat each response as a directly observed variable working independently. Instead, each physiological parameter can be used as a composite latent variable describing overall homeostatic disturbance. That is, each individual parameter contributes to a higher order construct, but they do not cause any motivational disturbances on their own. This latent factor approach is common in psychology but would be physiologically innovative and align with the idea that system‐wide responses to exercise manifest into an overall gauge of homeostatic integrity.^5^

Future research should also examine how to delay the desire‐goal conflict, particularly when the desire begins to overcome the performance goal value. Delay can be achieved by lowering the desire to reduce effort, increasing the magnitude of the performance goal value, or a combination of these strategies. Existing work has suggested that enhancing the congruence between the performance goal and one's identity^12^ can disturb the desire‐goal conflict favorably. This approach fits with other motivational frameworks, such as the identity‐value model^40^ and self‐determination theory.^41^ Other strategies have potential, such as enhancing the congruence between actively managing discomfort and the performance goal (i.e., means‐end fusion^42^).

### 4.2 Perspective

The desire‐goal conflict is a recently applied motivational framework that helps explain endurance performance.^10^, ^12^ Unlike most other models of endurance that considers motivation as a unidimensional construct,^1^ motivation is viewed as a network of constructs and related processes. Moreover, it has the potential to reconcile different physiological and psychological ideas, such as the role of physiological responses to exercise and their affective labels in shaping motivational processes.^3^, ^4^ Overall, the present study represents the first analysis of how the desire‐goal conflict is disturbed by two physiological thresholds (i.e., LT1 and LT2, and how these delineate fundamental exercise intensity domains). These thresholds, therefore, represent important points when motivational factors become increasingly detrimental to performance and intervention is required. In addition, analyzing within‐person variation in blood lactate and heart rate may portray a clearer picture of their relationship with motivation constructs, compared with absolute levels.

## Data Availability

The data that support the findings of this study are available from the corresponding author (IT), upon reasonable request.
